# Generator based approach to analyze mutations in genomic datasets

**DOI:** 10.1038/s41598-021-00609-8

**Published:** 2021-10-26

**Authors:** Siddharth Jain, Xiongye Xiao, Paul Bogdan, Jehoshua Bruck

**Affiliations:** 1 California Institute of Technology, Electrical Engineering, Pasadena, 91125 USA; 2 University of Southern California , Electrical and Computer Engineering, Los Angeles, 90007 USA

**Keywords:** Computational biology and bioinformatics, Mathematics and computing

## Abstract

In contrast to the conventional approach of directly comparing genomic sequences using sequence alignment tools, we propose a computational approach that performs comparisons between sequence generators. These sequence generators are learned via a data-driven approach that empirically computes the state machine generating the genomic sequence of interest. As the state machine based generator of the sequence is independent of the sequence length, it provides us with an efficient method to compute the statistical distance between large sets of genomic sequences. Moreover, our technique provides a fast and efficient method to cluster large datasets of genomic sequences, characterize their temporal and spatial evolution in a continuous manner, get insights into the locality sensitive information about the sequences without any need for alignment. Furthermore, we show that the technique can be used to detect local regions with mutation activity, which can then be applied to aid alignment techniques for the fast discovery of mutations. To demonstrate the efficacy of our technique on real genomic data, we cluster different strains of SARS-CoV-2 viral sequences, characterize their evolution and identify regions of the viral sequence with mutations.

## Introduction

Phylogenetic approaches have had enormous success in explaining and visualizing the temporal and spatial evolution of different species. The methods used for constructing phylogenetic trees rely on either calculating edit distance or other distances which are based on a detailed evolutionary model^1^. There have been several approaches to compute the edit distance and perform sequence alignment in the past^2–4^. One of the main purposes of pairwise and multiple sequence alignment methods is the discovery of mutations (substitutions, indels) and the differentiation of sequences being compared. In this paper, we provide an alternative method that performs sequence comparisons based on the difference between the rules that may be used to generate sequences. More precisely, we hypothesize that genomic sequences have an underlying generator which can be characterized by a fixed number of parameters. Any genomic sequence that we observe in nature is an instance of this parametric generator. From a computational perspective, these sequence generators provide us with an efficient way of compressing genomic information which further allows us to make statistical comparisons amongst large genomic datasets and can be used to cluster genomic sequences, characterize temporal and spatial evolution in a continuous manner, detect local sites with higher mutation activity, as well as to aid alignment techniques for fast and efficient discovery of mutations. As a case study, we further demonstrate these aforementioned advantages on different strains of the SARS-CoV-2 virus.

**Figure 1:**
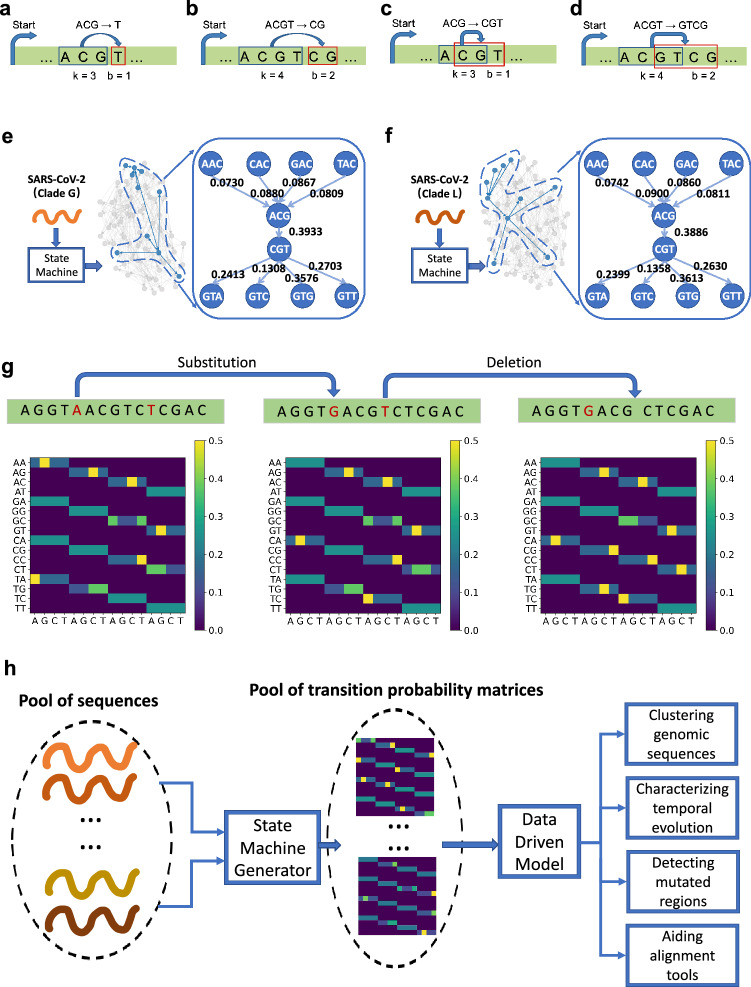
**Overview of the method.** The generator is based on a state machine. A genomic sequence is mapped onto a set of states and transition probabilities. The states are identified by substrings of length *k* and the transition probabilities are computed by observing the next *b*-length substrings following the current state (see (**a**,**b**)). Another way to understand the model for $k = 3, b = 1$ and $k = 4, b = 2$ is shown in (**c**,**d**) respectively. These state machines can also be viewed as weighted directed graphs where nodes represent the states and the edge weights correspond to the transition probabilities. In (**e**,**f**), we show the weighted directed graph characterization of SARS-CoV-2 sequences belonging to GISAID Clade G and Clade L through the state machine model $k = 3, b = 1$. In (**g**), we show the transition probability matrix for the state machine with $k = 2,$ $b =1$ and illustrate the impact of a single substitution and a single deletion on the transition probability values. (**h**) shows the flow diagram of the proposed method.

We map a genomic sequence onto a generator modeled using a state machine, i.e., a directed weighted network where the nodes and edges represent the states (i.e., contiguous groups of *k* nucleotides) and the transition probabilities among states, respectively (see Fig.1c,d). In order to predict the next *b* nucleotides from previous *k* contiguous nucleotides in the genomic sequence, we develop a Markov chain consisting of $4^k$ states transitioning into $4^b$ states (see Fig. 1a,b). After traversing the entire sequence, the number of occurrences of each transition provides information about the corresponding transition probability and also represents the weight of the corresponding directed edge (i.e., a directed weighted network of the RNA sequence is generated with $4^k$ nodes (see Fig. 1e,f)). Figure 1a,b show a transition $acg \rightarrow t$ with model $k = 3,~ b = 1$. Figure 1c,d show a transition $acgt \rightarrow cg$ with model $k = 4, ~b = 2$. This proposed method encodes the generation of genomic sequences and the transition probabilities reveal important context-sensitive characteristics within the sequence. The state machine based generator is independent of the sequence length and the number of states. The transitions are model parameters that can be set by the user. The non-reliability of the sequence length implied by our technique makes the method less susceptible to misalignment issues caused by deletions and insertion mutations in genomic sequences.

Next, we discuss the efficacy of sequence generator approach based on the state machine model for different genomic sequence based applications. In particular, we focus on (*i*) clustering large genomic sequence datasets, (*ii*) characterizing the temporal evolution of genomic sequences in a continuous manner, (*iii*) proposing a *data-driven* mutation region detection algorithm that can be used to infer local statistics within a sequence to give insights about the mutation activity within the genomes, and (*iv*) using the proposed mutation region detection algorithm to aid the state of the art alignment techniques for faster discovery of mutations.

**Figure 2:**
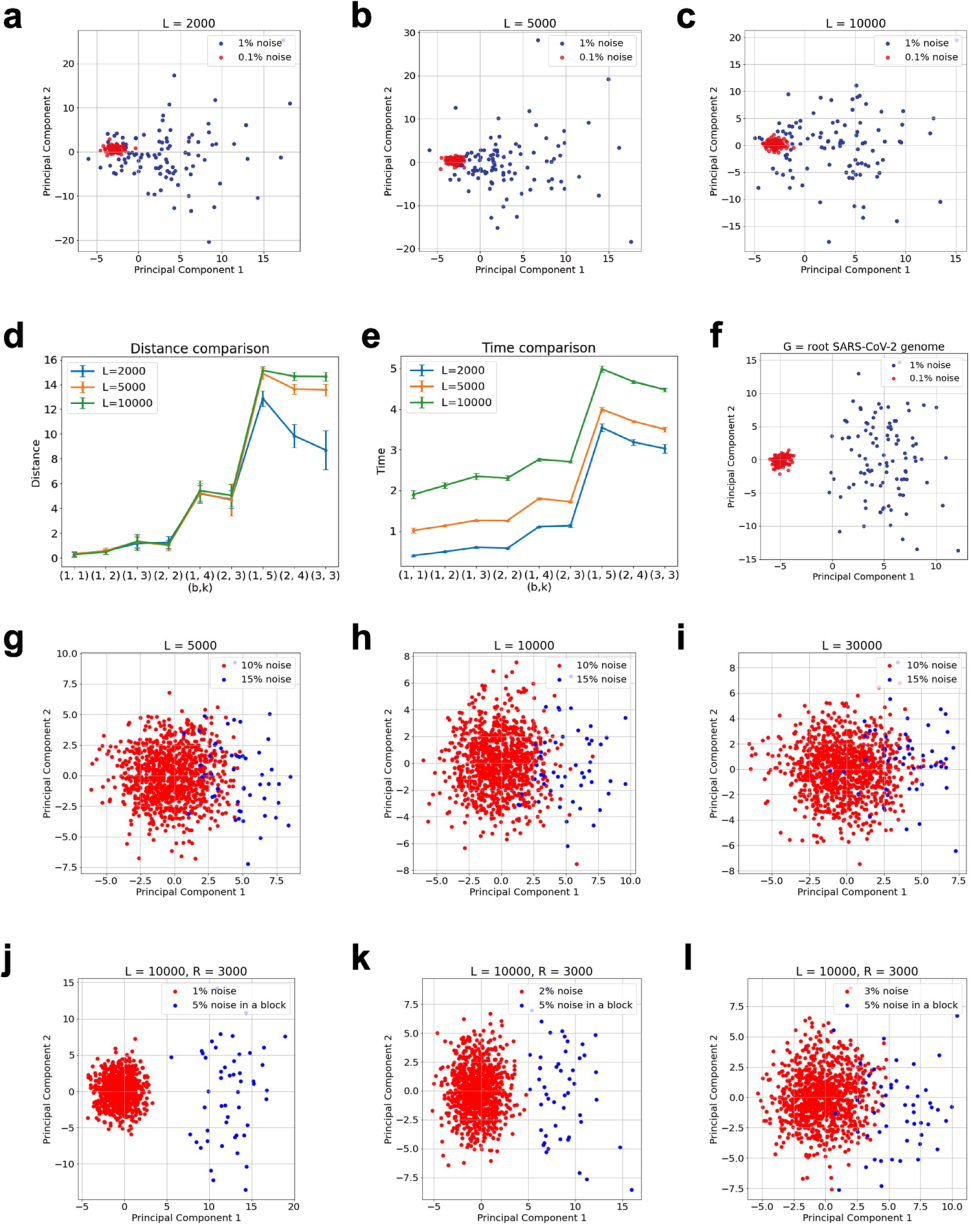
**Clustering of Sequences.** **(a–e)** Red and blue sequences are generated by adding substitution, insertion and deletion noise with probability $0.1\%$ and $1\%$ respectively. (**a**–**c**) show the first 2 principal components as a result of applying PCA on the state machine representations of the sequences with $k = 4, b = 1, \beta = 0.5.$ for $L = 2000, 5000, 10000$ respectively. (**d**) shows distances between the centers of the red and blue clusters generated by different combinations of *b* and *k*, while (**e**) shows the time complexity of the method with different combinations of *b* and *k*, we repeat 20 times in each experiment and show the mean and standard deviation. **(f)** Red and blue sequences are generated by adding substitution, insertion and deletion noise with probability $0.1\%$ and $1\%$ respectively on the root SARS-CoV-2 sequence. (**f**) illustrates similar clustering performance for $k = 4, b = 1, \beta = 0.5$ as observed in (**a**–**c**) when noise is applied on a real sequence, i.e. SARS-CoV-2. (**g–i**) Red and blue sequences are generated by adding substitution, insertion and deletion noise with probability $10\%$ and $15\%$ respectively. (**g**–**i**) show the first 2 principal components as a result of applying PCA on the state machine representations of the sequences with $k = 4, b = 1, \beta = 0.5.$ for $L = 5000, 10000, 30000$ respectively. (**j–l**) The red sequences have a noise level of $x\%$ and are of length $L = 10000.$ The blue sequences of length $L = 10000$ have a noise level of $x\%$ in locations $1-1000$ and $4001-10000$, and a noise level of $5\%$ in locations $1001-4000$. (**j**–**l**) show the first two principal components of applying PCA on the state machine representation (with $k = 4, b = 1, \beta = 0.5$) of red and blue sequences for $x = 1, 2, 3$ respectively.

**Figure 3:**
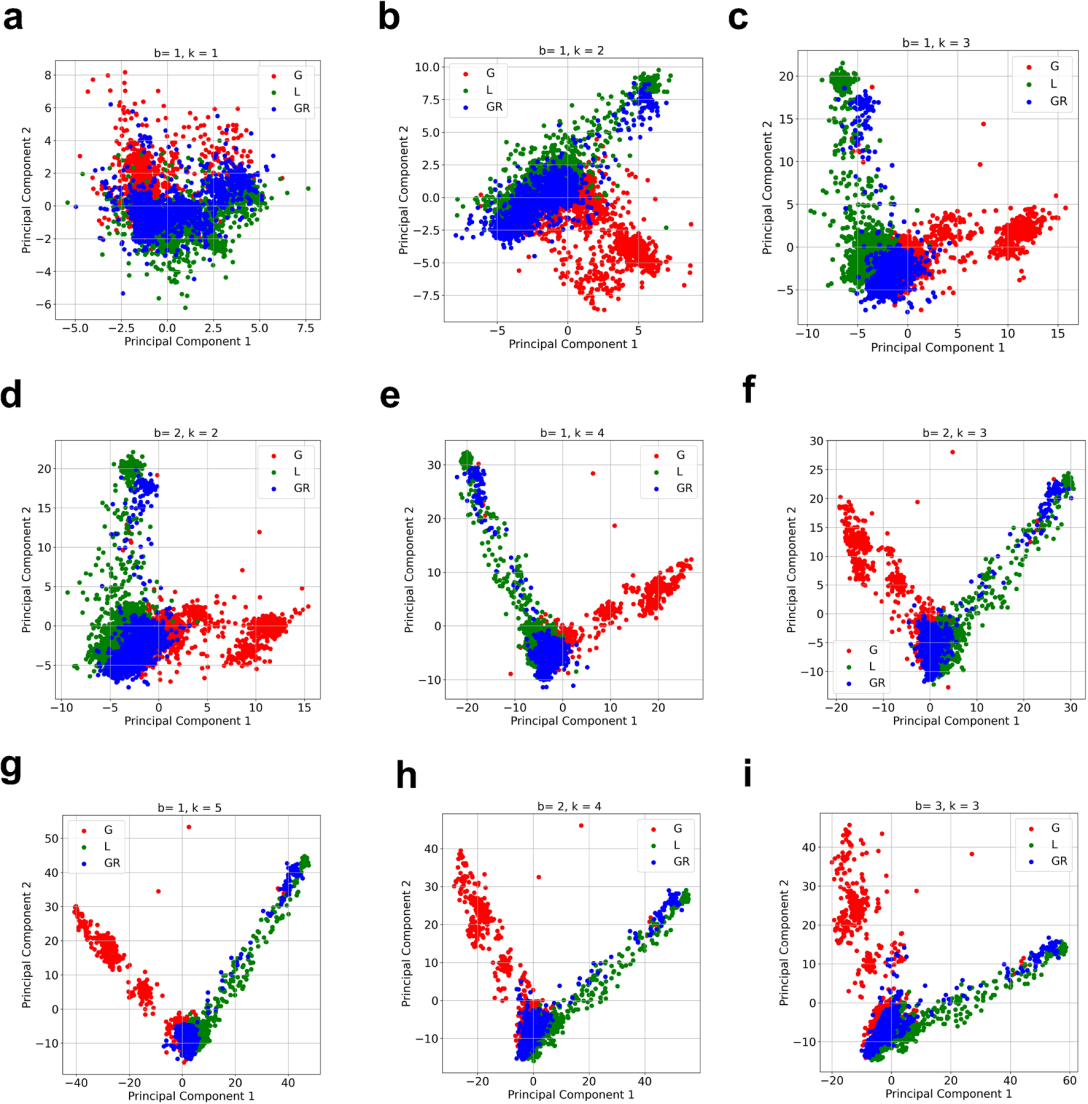
**Clustering of SARS-CoV-2 Clades.** (**a**–**i**) Clustering using PCA of GISAID clades L, G and GR of SARS-CoV-2 using the state machine representation for different values of *b* and *k* with $\beta = 0.5.$ As can be seen, the clustering performance is better when $k+b$ is larger for example $b= 1, ~k = 4 ~(k+b = 5)$, shows a better clustering performance then $b = 1,~ k = 2 ~(k+b = 3).$ The performance for $b = 1, ~k = 5$ or $b= 2, ~k = 4$ or $b = 3, ~k = 3 ~(k+b = 6)$ is near similar when compared to $b = 1, ~k=4$ or $b =2, k = 3~(k+b=5)$, however the computational time is more when $k+b$ is larger as shown in Fig. 2. Hence for this example, $b = 1, ~k= 4$ or $b = 2,~ k =3$ is a good choice of parameters as it optimizes both clustering performance and computational time.

## Results

### The inferred state machine generator can cluster genomic sequences and characterize their temporal evolution

#### Sequence clustering

The sequence generators inferred for every sequence using the proposed state machine model can be used to cluster sequences, hence, identifying sequences with similar and distinguishable generators. We conduct different experiments on synthetic and real datasets to show the efficacy of the model in clustering genomic sequences. In the first experiment, we generate synthetic genomic datasets. We first generate a synthetic genomic sequence $G$ of length *L*, the sequence is generated randomly. Next, we obtain noisy copies of $G$ by adding stochastic noise at each location in $G$. The noise can be either a substitution, insertion or deletion with equal probability. Let ${\mathscr {A}}$ and ${\mathscr {B}}$ denote the sets of noisy sequences obtained from $G$ using probabilities $p_{{\mathscr {A}}}$ and $p_{{\mathscr {B}}}$, respectively. Let ${\mathscr {A}} = \{A_1,A_2,\cdots ,A_u\}$ and ${\mathscr {B}} = \{B_1,B_2,\cdots ,B_v\}.$ Given a pair of *k* and *b* parameters, we obtain the data-matrix $\pmb {X}$ of size $(u+v)\times 4^{k+b}$ for $A_1,A_2,\cdots ,A_u,B_1,B_2,\cdots ,B_v$ (as described in Methods section). Fig. 2a–c show the results of applying principal component analysis (PCA) on data matrix $\pmb {X}$ obtained by the approach discussed in Methods section for $k = 4$, $b = 1$, $\beta = 0.5$, $u = 100$, $v = 100$, $p_{{\mathscr {A}}} = 0.001$ and $p_{{\mathscr {B}}} = 0.01$ for $L=2000,5000,10000$. We notice that the sequences in sets ${\mathscr {A}}$ and ${\mathscr {B}}$ can be clustered for different values of *L*. To observe the impact of choosing parameters *k* and *b* on clustering performance, we use different combinations of *k* and *b* to conduct the above experiments and calculate the distance between the centers of two clusters (i.e., ${\mathscr {A}}$ and ${\mathscr {B}}$), the results are shown in Fig. 2d. Figure 2e shows the time complexity of the methods with different combinations of *k* and *b*. The distance represents the ability of the method to distinguish two clusters, as shown in Fig. 2d,e, when $(k+b)$ becomes higher, both the distance and time complexity become higher. When $(k+b)$ jumps from 5 to 6, both the distance and the time complexity increase significantly. In Fig. 2f, we conduct the same experiment (with $k = 4, b = 1, \beta = 0.5$) as done to generate Fig. 2a–c but by choosing the starting sequence *G* as SARS-CoV-2. We observe similar clustering performance (as in Fig. 2a–c) in Fig. 2f when *G* is chosen as a real sequence, i.e. SARS-CoV-2. In Fig. 2g–i, we also show the results of applying PCA on data matrix $\pmb {X}$ obtained using the approach discussed in Methods section for $k = 4$, $b = 1$, $\beta = 0.5$, $u = 1000$, $v = 50$, $p_{{\mathscr {A}}} = 0.1$ and $p_{{\mathscr {B}}} = 0.15$ for $L=5000,10000,30000$. We can see that the sequences in sets ${\mathscr {A}}$ and ${\mathscr {B}}$ can be clustered for different values of *L*. Further, the number of samples in set ${\mathscr {A}}$ ($u = 1000$) is chosen to be much larger than the number of samples in set ${\mathscr {B}}$ ($v = 50$) in generating Fig. 2g–i to show the efficacy of the method in identifying rare clusters. Further in Figure S1, we conduct another experiment where we show that the method can cluster sequences from a sequence pool where all sequences have the same mutation probability *p* but can have different starting sequences.

In the second experiment on synthetic data, we again consider a sequence $G$ of length *L*. Next, we obtain noisy copies of $G$ by adding stochastic noise at each location in $G$. The noise can represent either substitution, insertion or deletion with equal probability. Let ${\mathscr {A}}$ denote the set of noisy sequences obtained from $G$ using probability $p = p_{{\mathscr {A}}}$. Let ${\mathscr {A}} = \{A_1,A_2,\cdots ,A_u\}$. Let ${\mathscr {B}} = \{B_1,B_2,\cdots ,B_v\}$. Here, $B_i$ is obtained by adding noise with probability $p_{{\mathscr {B}}}$ on a substring of length *R* in $A_{u-v+i}$ starting at a fixed index *j* and ending at the index $j+R-1$. For a pair of *k* and *b* values, we obtain the data-matrix $\pmb {X}$ of size $u\times 4^{k+b}$ for $A_1,A_2,\cdots ,A_{u-v},B_1,B_2,\cdots ,B_v$ (see Methods section). Figure 2j–l shows the result of applying PCA on data matrix $\pmb {X}$ obtained using the approach discussed in Methods section for $k = 4$, $b = 1$, $\beta = 0.5$, $u = 1000$, $v = 50$, $L = 10000$, $R = 3000$ and $j = 1001$ for different values of $p_{{\mathscr {A}}}$ and $p_{{\mathscr {B}}}$. The results show that the characteristics of the sequences in ${\mathscr {B}}$ can be captured by the state machine and then the difference between the sequence clusters with different mutation probabilities can be captured by PCA analysis. The distinction increases when the difference between the two clusters becomes greater. In one sequence cluster, the higher the mutation probability, the higher the divergence of the first two principal components.

Next, we show how the proposed method can distinguish between different SARS-CoV-2 clades and strains. In Fig. 3, we apply the state machine based generator to different SARS-CoV-2 clades and strains observed in year 2020. In Fig. 3, we show that the state machine representation can be used to cluster GISAID clades G (1818 samples), L (1732 samples) and GR (1622 samples)^5^ using the first two principal components of PCA analysis. Further, we show the impact of choosing parameters *k* and *b* on clustering performance. As can be observed from Fig. 3, the clustering performance is improved as $k+b$ is increased. When $k+b\ge 5$, we observe good quality clustering between GISAID clades G, L and GR using the proposed method (Note that $\beta = 0.5$ for all the experiments conducted in the paper.).

The feature weights on both the principal components and the important features when $k = 4, ~b= 1$ and $k = 3, ~b =3$ are shown in Supplementary Figures S4-S6. In Figure S2, we show the application of our approach to cluster and identify the distance between the UK strain (318 samples), South African strain (100 samples) and Brazilian strain (47 samples).

**Figure 4:**
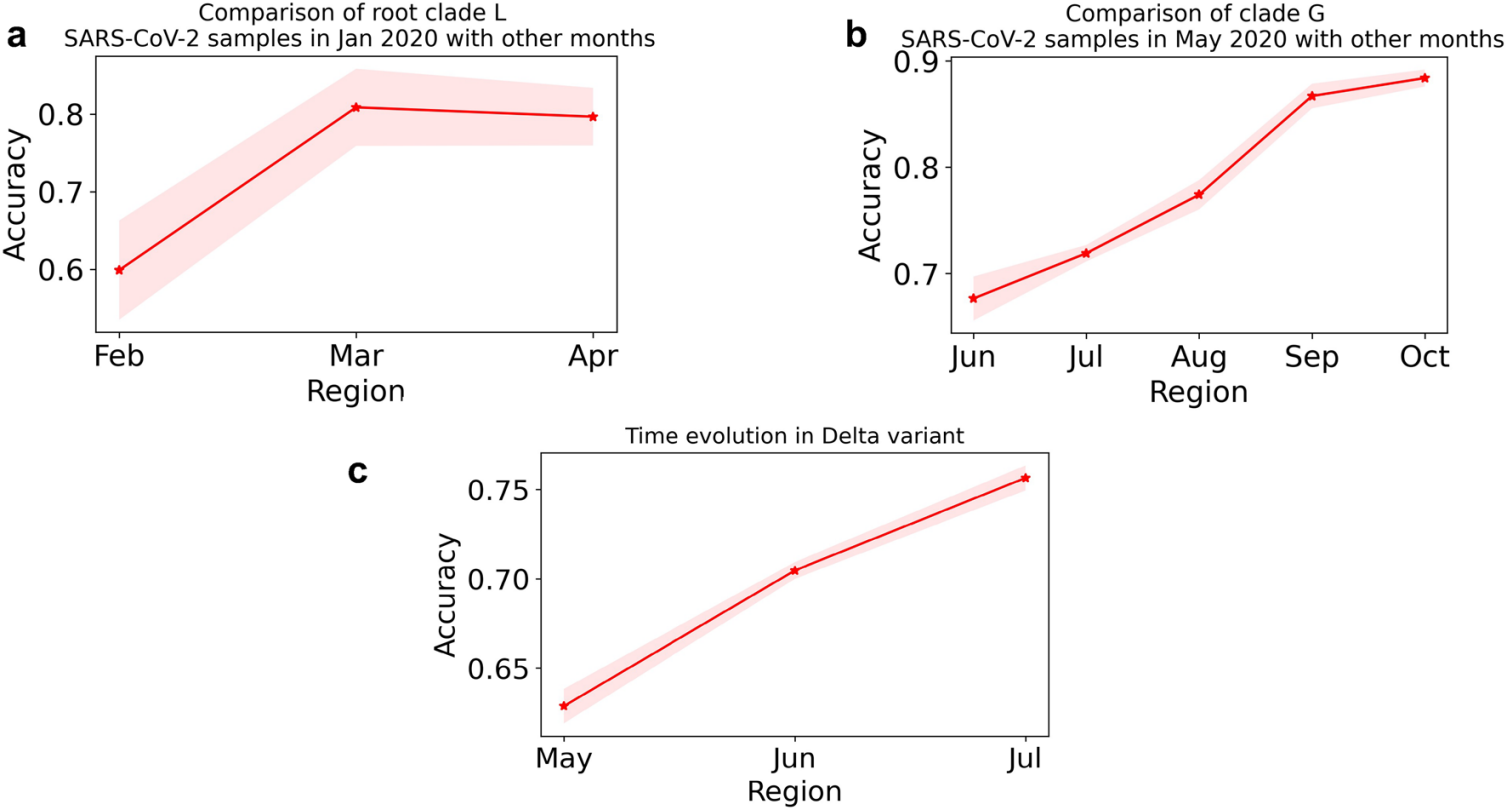
**Continuous Evolution.** A variant/strain is defined by deterministic stable mutations, however, evolution is characterized by the randomness of the transient mutations. In these plots, we quantify this randomness for different SARS-CoV-2 clades by comparing SARS-CoV-2 sequences within the same clade/variant in different months. (**a**) represents validation accuracy of a logistic regression classifier built using state machine representations of SARS-CoV-2 sequences (with $k = 4$, $b = 1$, $\beta = 0.5$) in January 2020 with the months of February, March and April 2020 within GISAID clade L. (**b**) represents validation accuracy of a logistic regression classifier built using state machine representations of SARS-CoV-2 sequences (with $k = 4$, $b = 1$, $\beta = 0.5$) in May 2020 with the months of June, July, August, September and October 2020 within GISAID clade G. (**c**) represents validation accuracy of a logistic regression classifier built using state machine representations of SARS-CoV-2 sequences (with $k = 4$, $b = 1$, $\beta = 0.5$) in Apr 2021 with the months of May, June, and July 2021 within Delta variant. Greater accuracy in both (a), (b) and (c) point towards more separation between the sequence classes being compared.

#### Characterizing temporal evolution in a continuous manner

Continuous evolution is characterized by quantifying the randomness of transient mutations. However, the clades/variants/strains are defined using stable mutations. In Fig. 4, we perform intra-clade and intra-variant comparisons for different months within SARS-CoV-2 and show the efficacy of our method in quantifying continuous evolution within SARS-CoV-2. More precisely, we make intra-clade comparisons for SARS-CoV-2 sequences for GISAID clades L and G over different months. In particular, let ${\mathscr {A}}$ and ${\mathscr {B}}$ denote the set of sequences categorized as clade L in months *A* and *B*, respectively. We obtain the data matrix $\pmb {X}$ using the sequences for months *A* and *B* and then build a logistic regression based classifier to compare month *A* and *B* sequences using $\pmb {X}$. The pairwise validation accuracy of the classifier can be used to measure the distance between sequences compared over different months (see Machine learning section in Methods section). Figure 4a shows the results of comparison when 209 clade L samples from January 2020 are compared with clade L samples from February (209 samples), March (1739 samples) and April (788 samples), respectively. Figure 4b shows the comparison for 2433 clade G samples from May 2020 with clade G samples from June (2309 samples), July (1465 samples), August (1605 samples), September (2038 samples) and October (1818 samples), respectively. Figure 4c shows the comparison for 5699 Delta variant samples from April 2021 with Delta variant samples from May (4163 samples), June (5092 samples) and July (5284 samples), respectively. The corresponding heatmaps for the continuous evolution plots shown in Figure 4 are provided in Supplementary Figure S3. As can be observed from these results, the accuracies exhibit an increasing trend illustrating the power of the state machine representations to characterize continuous evolution within the same clade without any need for sequence alignment.

### The sequence generator can detect local regions with mutations in a sequence exploiting context sensitive information encoded by the state machine transition probabilities

We provide a data driven algorithm (see Methods section for the formal description of the algorithm) that can be used to detect the regions with mutation activity in the sequence using the state machine based representation. We also conduct different experiments on synthetic and real datasets to show the efficacy of the mutation region detection algorithm.

**Figure 5:**
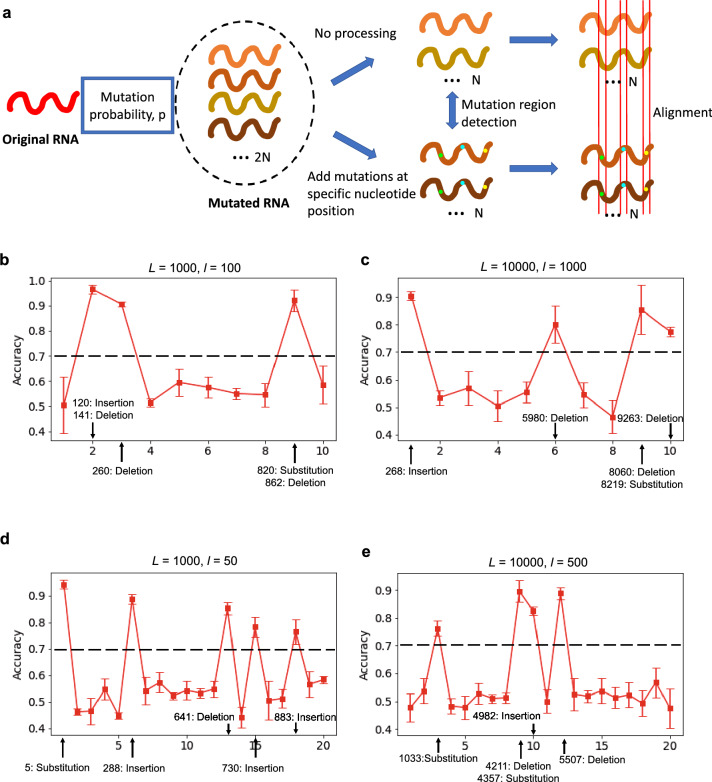
**Mutation Region Detection.** We use the synthetic experiments to show how our method works. (**a**) shows the process of the synthetic experiments. In the experiment, we first generate the original random RNA sequence of length *L*/*nt*, then we set the mutation probability of each nucleotide site on the original sequence to *p* to generate 2*N* mutated sequences. We assume that three common mutation types (i.e., substitution, insertion and deletion) occur with equal probability. Then we divide these 2*N* sequences equally into 2 groups, in the first group, we do not perform any processing, while in the second group, we randomly add the same new mutations to them (i.e., add the same mutation on the same nucleotide site). Then we use our mutation region detection algorithm (set the region length to *l*/*nt*) to detect the regions that have mutations, and apply the alignment algorithm on these regions to find the specific mutation. (b-e) We also take the examples with 4 different combinations of sequence length *L* and region length *l*: (**b**) $L=1000, l=100$; (**c**) $L=10000, l=1000$; (**d**) $L=1000, l=50$; (**e**) $L=10000, l=500$. We set the mutation probability *p* to $5\%$, and the number of sequences in each group to 100, then we randomly add 5 mutations on the sequences in the second group, and detect the mutation region by using our mutation region detection algorithm.

We first conduct an experiment on synthetic genomic data. Consider a sequence $G$ of length *L*. We obtain $2N (N=100)$ noisy copies of $G$ by adding stochastic noise at each location in $G$. The noise can be either substitution, insertion or deletion with equal probability. Let ${\mathscr {C}} = \{C_1,C_2,\cdots ,C_{2N}\}$ denote the set of noisy sequences obtained from $G$ using probability $p_{{\mathscr {C}}}$. We split ${\mathscr {C}}$ into 2 sets ${\mathscr {U}}$ and ${\mathscr {V}}$ such that ${\mathscr {U}} = \{C_1,C_2,\cdots ,C_N\}$ and ${\mathscr {V}} = \{C_{N+1},C_{N+2},\cdots ,C_{2N}\}.$ Consider a set of locations ${\mathscr {G}} = \{g_1,g_2,\cdots ,g_n\}$ and mutations ${\mathscr {M}} = \{m_1,m_2,\cdots ,m_n\}$. For each $C_j \in {\mathscr {U}}$ ($j \in \{1,2,\cdots ,N\}$), we now implant mutation $m_i$ at location $g_i$ for all $i \in \{1,2,\cdots ,n\}$ to obtain $C_j'.$ Let ${\mathscr {U}}'= \{C_1',C_2',\cdots ,C_N'\}$ denote the set of sequences with implanted mutations. Figure 5 shows the results of applying the mutation site detection algorithm on sequences in sets ${\mathscr {U}}'$ and ${\mathscr {V}}$ for different values of *L*, *l*, ${\mathscr {G}}$, ${\mathscr {M}}$ with $b = 1$, $k = 4$, and $\beta = 0.5$. These results show that all the mutated regions are captured by our algorithm, which can help us to find the specific mutation sites.

**Figure 6:**
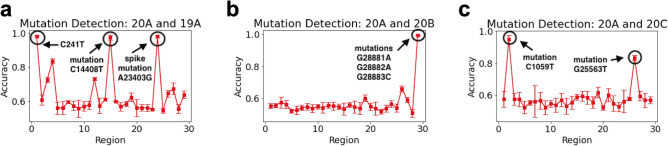
**Mutation region detection in SARS-CoV-2 clades.** Here, we show how the state machine model can be used to detect the mutations differentiating various clades without the need for sequence alignment. In this method, we divide each SARS-CoV-2 RNA sequence into 30 regions of length 1000 each and calculate the classification accuracy for the model comparing the transition probability matrix calculated for each 1000 length region. The regions for which the classification accuracy is high are the spots where the transition probability matrices are different due to mutations occurring in that region. For example, an accuracy of 1 in region 24 (23000–24000) in (**a**) indicates the detection of spike protein mutation (A23403G or D614G) in nextstrain clade 20A when compared against 19A. Further in plots (**b**,**c**), we detect regions with mutations differentiating clade 20A with 20B and 20C.

We also apply our algorithm on SARS-CoV-2 genomic data to detect the regions with mutations in different clades. The SARS-CoV-2 genomic data is obtained from GISAID^5^. We consider sequences in the nextstrain^6^ clades - 19A, 20A, 20B and 20C. The length *L* of SARS-CoV-2 RNA is $\sim 30,000$. We divided the SARS-CoV-2 RNA into non-overlapping regions of length $l = 1000$ each. Therefore, we have 30 regions. We now obtained the data matrix $\pmb {X}_i$ with $k = 4$, $b= 1$, $\beta = 0.5$ for each region $i \in \{1,2,\cdots , 30\}$ using the set of sequences in clade 19A (558 samples) and 20A (605 samples). Next, we design 30 logistic regression based classifiers for comparing clade 19A and 20A sequences in the 30 regions. We notice high pairwise accuracy for classifiers in regions where mutations differentiating clades 19A and 20A are present (see Fig. 6). We apply the same technique for comparing 20*A* with other nextstrain clades—20B (561 samples), 20C (232 samples) (see Fig. 6). Therefore, our method can efficiently help in processing a large number of genomic sequences to detect the regions with mutations in different clades.

By using the region length *l* as a parameter, the proposed mutation region detection algorithm can be used to detect regions of mutations differentiating the two sets of sequences. Further, the algorithm can be used to aid alignment techniques as it can identify regions with mutations. In particular, having information about areas of mutations will significantly reduce the length of the sequences needed to be aligned to accurately identify the mutations saving computational time. We elaborate more on this insight next.

### Mutation region detection algorithm can be used to aid alignment techniques to discover mutations

Due to the state machine based representation, our mutation region detection method can efficiently process large sets of genomic sequences. In this part, we show how the mutation region detection method we proposed above can be used to aid alignment techniques and show faster performance compared to the state of the art alignment techniques.

State-of-the-art methods for detecting mutations in gene sequences rely on the sequence alignment algorithm. Moreover, the time complexity of these state-of-the-art alignment algorithms is O($L^2$)^7^, where *L* is the length of the sequence. Our proposed method can detect the mutation region in linear time and can be used to aid the alignment method. We first use our mutation region detection algorithm to find the region of the mutation, and then find the specific mutation through sequence alignment of the specific region between the sequences. In this way, the time complexity of mutation detection will be significantly reduced.

**Figure 7:**
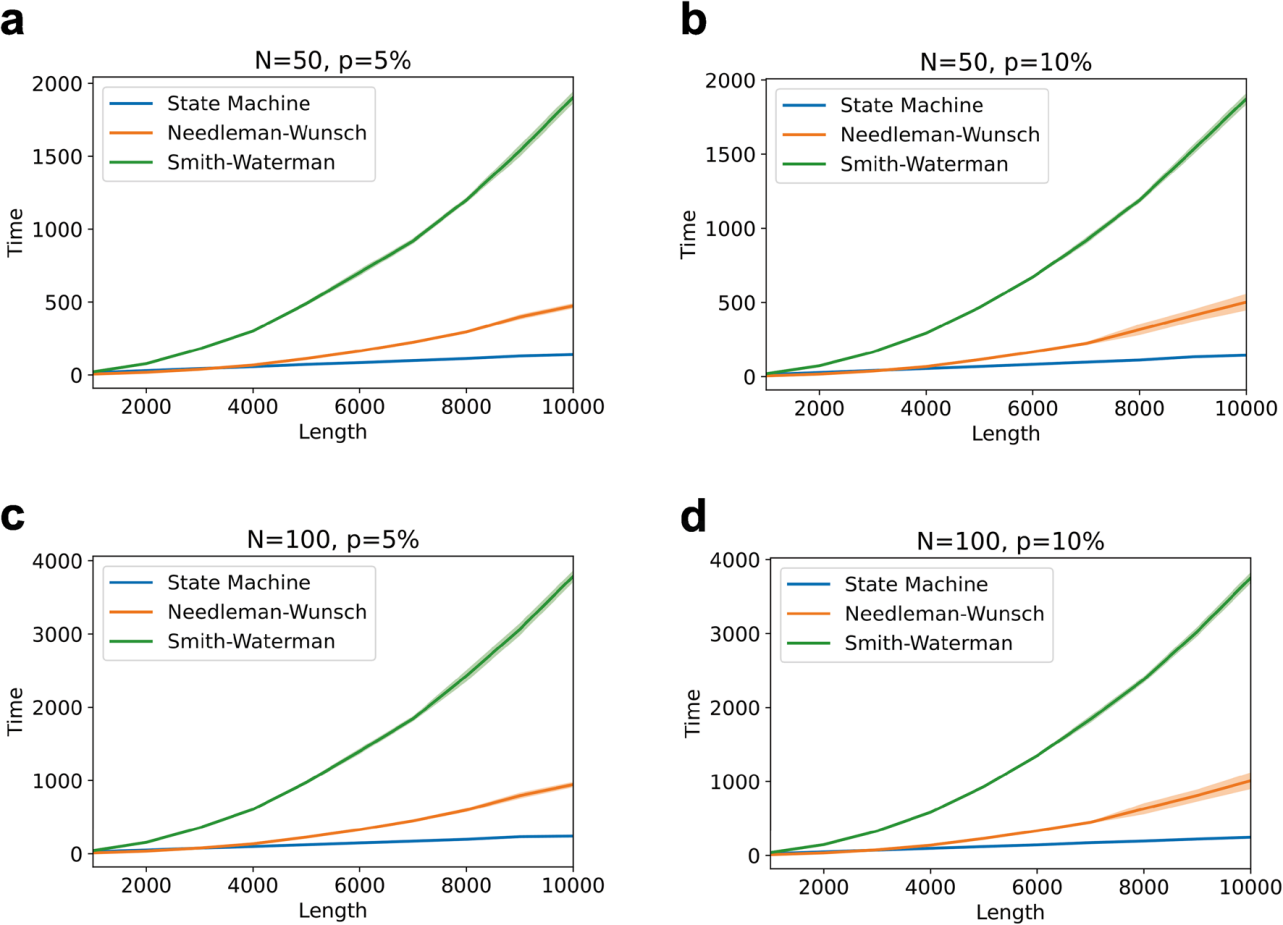
**Time complexity analysis. **We compare the running time of our mutation detection method and two common alignment algorithm through the synthetic experiments. We use 4 combinations of different number of sequences in each group *N* and mutation probability *p*: (**a**) $N=50, p=5\%$; (**b**) $N=50, p=10\%$; (**c**) $N=100, p=5\%$; (**d**) $N=100, p=10\%$. The time complexity of our method is *O*(*N*) while the time complexity of the state of the art alignment algorithms is O($N^2$). The biopython package implementation of Smith–Waterman and Needleman-Wunsch algorithms were used in our analysis. As can be seen, the proposed method has clear time complexity advantages for the sequences longer than 4000/*nt*. The system configuration of the computer on which the experiments were run are: Processor: 2.4 GHz Intel Core i5; Memory: 16 GB 2133 MHz LPDDR3.

We conduct experiments on synthetic genomic data to show the difference in terms of time complexity between our method and the biopython package implementation of 2 popular sequence alignment algorithms (i.e., the Needleman-Wunsch and Smith-Waterman algorithms). In the synthetic experiment (see Fig. 5), we set the number of sequences in each group to *N*, the mutation probability to *p*, and we use 4 different combinations (i.e., $N=50$ and $p=5\%$, $N=50$ and $p=10\%$, $N=100$ and $p=5\%$, $N=100$ and $p=10\%$) to test the time the method takes to detect the mutations. In the state machine method, we first use our mutation region detection algorithm to find the mutation region, and in the second stage, we use the Smith-Waterman algorithm to detect the specific mutations. We record the total time of these two steps. As is shown in Fig. 7, the approach where our method is used in the first step empirically runs in total linear time while the time complexity of the Needleman-Wunsch algorithm and Smith-Waterman algorithm is quadratic^7–9^ in sequence length. In practice, for sequences longer than 4000*nt*, our state machine method shows clear advantages over the other two algorithms (see Fig. 7). A more precise analysis of the time and space complexity of our algorithm is provided in the Methods section section.

## Discussion

We propose a data driven approach inspired by Kolmogorov complexity to infer and model generators of genomic sequences. We conceptualize the sequence as being generated by a state machine (Fig. 1). This new modeling perspective provides not only an alternative method to analyze and statistically compare large datasets of genomic sequences without any need of alignment, but also opens new analytical and practical directions of scientific inquiry. The method can be exploited to (*i*) cluster different sequence strains (Fig. 2) (*ii*) characterize the evolution in a continuous manner (Fig. 4), (*iii*) infer the local statistics within a sequence (Figs. 5, 6) , and (*iv*) aid alignment techniques for faster discovery of mutations (Fig. 7).

By characterizing the higher order interactions among the RNA/DNA nucleotides within the sequence, we provide new mathematical tools for investigating the evolution of genomic sequences. Our work also enables the development of novel approaches to transform sequences into new representations which preserve the distance between the sequences, store contextual information and are robust to insertion, deletion and substitution mutations. Another important characteristic of the state machine model is that it can be used to capture the scale dependencies among various regions of the nucleotide sequences through the transition probabilities.

The proposed sequence generator based on a state machine model can be viewed as a universal and interpretable tool to apply computational analysis on genomic sequences. Higher-order interactions among the nucleotides could be computationally analyzed and used to characterize the genomic sequences, providing an approach to investigate sequences at the genetic level. As shown in the paper, our model can be used to compare between different sequence strains and detect regions with mutations separating these strains (Fig. 6).

We also capture the temporal characteristics of sequence evolution. In particular, the model can be used to characterize continuous evolution within a clade (Fig. 4). This statistical approach also allows us to quantify the effects of transient mutations which go back and forth during the temporal evolution of genomic sequences. Further, the mutation region detection algorithm infers the local statistics of genomic sequences in the dataset. Therefore, it can also be used to identify actively mutating areas in the genome. For example, one can compare state machines of local regions at different points in time and use the validation accuracy of the classifiers to identify local areas with higher mutation rates compared to others (see Fig. 6). An insight into local regions with high mutation activity can point us to areas that are being selected if the founder effect considerations are taken into account carefully^10^.

The temporal analysis of genomic sequences has been primarily focused on finding the phylogeny based on a model which considers computing pairwise edit distance between sequences^1^. A model based on the frequency of *k*-mers was proposed in the past for alignment free and global analysis of RNA and DNA sequences^11^. This model was recently used in^12^ to compare SARS-CoV-2 sequences with other viruses and earlier SARS-CoV-2 samples with the more recent ones. Recently, a *k*-mer based alignment-free classification approach was also used to discover viral type sequences within the human genome. The striking finding here is the evidence that viral genomes are dissimilar to human genome fragments if the sequences are aligned, but are rather similar in their *k*-mer distributions when compared to *k*-mer distributions in human genome^13^. In this paper, we present a sequence generator based on state machine for analyzing genomic sequences. Similar to *k*-mer frequency based approach^11^, this model also doesn’t require alignment of sequences. Moreover, the approach allows us to make statistical comparisons between large sets of genomic sequences in a fast and efficient manner. Further, it can be used to characterize evolution, detect local regions with mutations which can then be used to aid alignment techniques for mutation discovery in those regions.

Biologists can take our model and use it as a universal graph representation for all the genomic sequences. The nodes in the graph represent the states and the edges are defined by the state transitions. The graph is universal as the graph structure is independent of the sequence of interest. The weighted graph structure encodes the specific higher-order interactions within a nucleotide sequence and can be used to define the distance between different sequence types. Further, this mathematical framework can also be used to infer the states in the state machine which are less random (low entropy rate) compared to the ones which are random (high entropy rate). The low entropy states in the state machine are more predictable as they have higher probabilities for certain transitions over the others giving us insights on the context sensitive information in genomic sequences.

Our method also provides us with a preprocessing tool to aid alignment techniques. The preprocessing step comprises of the mutation region detection algorithm to identify local regions of smaller length with mutations and then uses state-of-the-art alignment tools to discover mutations saving computational time (see Fig. 7). We must point out here, that the performance of the mutation region detection algorithm can be limited by large blocks of deletion and insertions in the sequence, as large insertions and deletions can lead to significant differences in the local regions being compared. A possible solution to tackle this limitation is to modify the mutation region detection algorithm by allowing overlapping local regions. The amount of overlap needed and its formal analysis given the largest deletion and insertion in the sequences is a direction of our future work.

## Methods

We propose an alternative approach to perform comparison of genomic sequences which comprises two steps:

1. *Transformation:* We first transform the genomic sequence into a state machine that encodes information about the generative model of the sequence.
2. *Comparison:* We then compute distance between these state machine representations obtained in Step 1 using a data-driven approach.

### Transformation

Given a sequence $S$ of length *L*, the sequence is transformed into a state machine with parameters *k* and *b*. There are $4^k$ states and the empirical transition probability matrix $\pmb {T}$ is estimated from *S* using the add-$\beta$ estimator^14,15^ given by

1 $$\begin{aligned} \pmb {T}_{ij} = {\left\{ \begin{array}{ll}\frac{N_{ij}+\beta }{N_{i}+4^b\beta }, ~j~is~a~neighbor~of~i.\\ 0,~ otherwise.\end{array}\right. } \end{aligned}$$

Here $i \in \{1,\cdots , 4^k\}$. *j* is a neighbor of *i* if the last $k-b$ symbols of *i* overlap with the first $k-b$ symbols of *j* (see Fig. 1). Therefore, there are $4^b$ neighboring *k*-mers of *i*. $N_{ij}$ denotes the number of occurences of *k*-mer *i* and its neighboring *k*-mer *j* together in the sequence (see Fig. 1). $N_i$ denotes the number of occurrences of *k*-mer *i* in the sequence. The obtained matrix $\pmb {T}$ has $4^k$ rows and $4^b$ columns. A row-by-row scan of matrix $\pmb {T}$ is performed to convert $\pmb {T}$ into a vector $\pmb {x}$ of size $4^{k+b}$.

The obtained vector $\pmb {x}$ is the transformation of sequence $S$ into a state machine. In the comparison step discussed next, $\pmb {x}$ is used to compare different genomic sequences. Note that the size of the vector $\pmb {x}$ is only dependent on the parameters *k* and *b* and is independent of the sequence length *L*.

The state machine based representation derived in the transformation step discussed above encodes the probability of seeing a given nucleotide after a given substring in the sequence. Therefore, it captures the context sensitive information within the genomic sequence. Another feature of this transformation is that the size of the state machine is independent of the sequence length and is only controlled by parameters *k* and *b*. This independence from sequence length *L*, gives us a method to perform alignment-free comparisons between genomic sequences as discussed next.

### Comparison

Given *n* sequences $S_1, S_2,\cdots ,S_n$, they are transformed to $\pmb {x}_1, \pmb {x}_2, \cdots , \pmb {x}_n$ respectively using the approach discussed in the Transformation subsection above for a given set of parameters *k* and *b*. Using the vectors $\pmb {x}_1, \pmb {x}_2, \cdots , \pmb {x}_n$, we now obtain a data matrix $\pmb {X}$ with $\pmb {x}_i$ denoting the $i{th}$ row of $\pmb {X}$ for $i \in \{1,2,\cdots ,n\}.$ Therefore $\pmb {X}$ is a $n\times 4^{k+b}$ size matrix. The matrix $\pmb {X}$ can now be used to perform any statistical data analysis required to compare different genomic sequences.

Note that unlike alignment based methods that require multiple sequence alignment of sequences $S_1, S_2,\cdots ,S_n$ to perform data analysis, the data matrix $\pmb {X}$ obtained by the proposed approach doesn’t require the alignment step and instead obtains a representation encoding the generative model of the given sequences. Further the time complexity of obtaining $\pmb {X}$ from $S_1,S_2,\cdots ,S_n$ is given by $O(\sum _{i=1}^nL_i)$, where $L_i$ is the length of $S_i$ for $i \in \{1,2,\cdots ,n\}.$

### Mutation region detection algorithm

*Partitioning method:* Consider a sequence *S* of length *L*. Let *l* be a positive number $\le \frac{L}{2}.$ Partition the sequence *S* into non-overlapping blocks $B_1,B_2,\cdots , B_{\lceil {\frac{L}{l}}\rceil }$, where $B_i$ is of length *l* for $i \in \{1,2,\cdots ,\lfloor {\frac{L}{l}}\rfloor \}$ and $B_{\lceil {\frac{L}{l}}\rceil }$ is of length $L-l\lfloor {\frac{L}{l}}\rfloor$ is *L* is not a multiple of *l*. Given $b,k,\beta$, obtain the state machine representation or the transition probability vector $\pmb {T}^i$ for each $B_i$. We describe this formally in Algorithm 1 below.

*Algorithm:* Given two sets of sequences ${\mathscr {A}} = \{A_1,A_2,\cdots ,A_N\}$ and ${\mathscr {B}} = \{B_1,B_2,\cdots ,B_N\}.$ For each sequence $a \in {\mathscr {A}}\cup {\mathscr {B}}$, apply the partitioning method to obtain transition probability vectors $\pmb {T}_a^i$ for $i \in \{1,2,\cdots , \lceil {\frac{L}{l}}\rceil \}.$ For each $i \in \{1,2,\cdots , \lceil {\frac{L}{l}}\rceil \}$, obtain the data matrix $\pmb {X}^i$ using $\pmb {T}_a^i$ as a row for all $a \in {\mathscr {A}}\cup {\mathscr {B}}.$ Now build a binary classifier $\pmb {C}^i$, using $\pmb {X}^i$ by labeling rows obtained from set ${\mathscr {A}}$ as 0 and ${\mathscr {B}}$ as 1. Obtain the validation accuracy of the classifier $\pmb {C}^i$. If for a given region *i*, the validation accuracy is high, then the region *i* has a mutation or a set of mutations differentiating sequences in ${\mathscr {A}}$ with ${\mathscr {B}}.$ We describe this formally in Algorithm 2 below. 
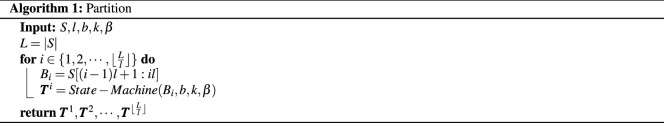

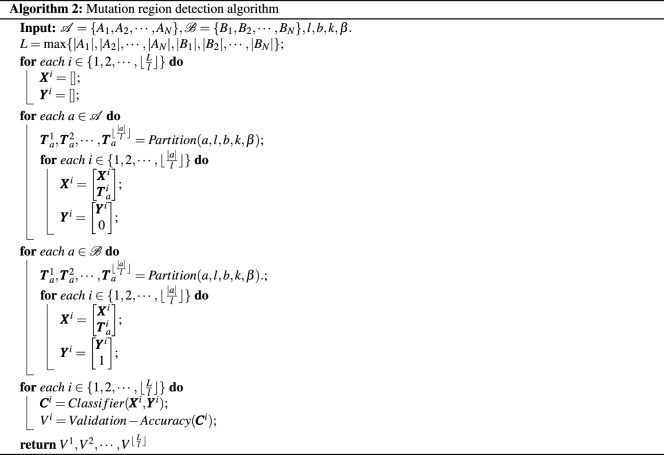


*Time complexity:* We notice here that the partition algorithm has a time complexity of $O(4^{k+b}\lfloor {\frac{L}{l}}\rfloor ).$ The overall time complexity of the mutation region detection algorithm therefore is given by $O(N4^{k+b}\lfloor {\frac{L}{l}}\rfloor +\lfloor {\frac{L}{l}\rfloor }t(C))$, where *t*(*C*) is the time complexity of the learning based classification algorithm.

*Space Complexity:* The auxiliary space complexity incurs due to additional storage of $2N\lfloor {\frac{L}{l}\rfloor }$ state machines of size $4^{k+b}$. Therefore the overall auxiliary space complexity is given by $O(N4^{k+b}\lfloor {\frac{L}{l}\rfloor }+s(C)\lfloor {\frac{L}{l}\rfloor })$, where *s*(*C*) is the space complexity of the classification algorithm.

### Statistical method used to compare genomic sequences

For a state machine model, the transition probability matrix is converted into a vector. We note that there can be at most $4^{k+b}$ non-zero entries in that vector as for any *k*-mer there are $4^b$ neighbors. We use this $4^{k+b}$ dimensional vector as the feature vector for building machine learning based classifiers. In all the classification tasks, we have 2 stages. In the first stage, we perform feature extraction by building a learning model using XGBoost^16,17^ (XGBoost package in python is used in the implementation) method with maximum depth = 1. In the next stage, we use the top 10 features identified in stage 1 to build a logistic regression based model for classification. We perform 4-fold cross validation in both the stages. While building these classifiers, the number of training samples for each class were equal to each other to account for data imbalance.

## Supplementary Information

Supplementary Information.

## Data Availability

The SARS-CoV-2 datasets analyzed in the current study are provided in GISAID (https://gisaid.org). The implementation of the method, acknowledgement files for the SARS-CoV-2 samples used and the necessary details to generate figures in the paper are provided at https://github.com/joshuaxiao98/Generator-based-approach-to-analyze-mutations-in-genomic-datasets. The acknowledgement files for the SARS-CoV-2 samples are also provided in the supplementary material.
