# Supplementary material for: Generator based approach to analyze mutations in genomic datasets
Source: Sci Rep. 2021 Oct 26;11:21084. doi: 10.1038/s41598-021-00609-8 (PMC8548350; doi:10.1038/s41598-021-00609-8)
Supplement: Supplementary file 1 — Supplementary Information. [file 41598_2021_609_MOESM1_ESM.pdf]

Supplementary Material for Generator based approach to analyze mutations in genomic datasets

Siddharth Jain[1+], Xiongye Xiao[2+], Paul Bogdan[2,*], and Jehoshua Bruck[1,*]

[1]California Institute of Technology, Electrical Engineering, Pasadena, 91125, USA
[2]University of Southern California , Electrical and Computer Engineering, Los Angeles, 90007, USA
[+]these authors contributed equally to this work
[*]pbogdan@usc.edu, bruck@caltech.edu

ABSTRACT

In contrast to the conventional approach of directly comparing genomic sequences using sequence alignment tools, we propose a computational approach that performs comparison between *sequence generators*. These sequence generators are learned via a *data-driven* approach that empirically computes the *state machine* generating the genomic sequence of interest. As the state machine based generator of the sequence is independent of the sequence length, it provides us with an efficient method to compute the *statistical* distance between *large sets* of genomic sequences. Moreover, our technique provides a fast and efficient method to cluster large datasets of genomic sequences, characterize their temporal and spatial evolution in a *continuous* manner, get insights into the *locality sensitive* information about the sequences *without any need* for alignment. Furthermore, we show that the technique can be used to detect local regions with mutation activity, which can then be applied to aid alignment techniques for *fast* discovery of mutations. To demonstrate the efficacy of our technique on real genomic data, we cluster different strains of SARS-CoV-2 viral sequences, characterize their evolution and identify regions of the viral sequence with mutations.

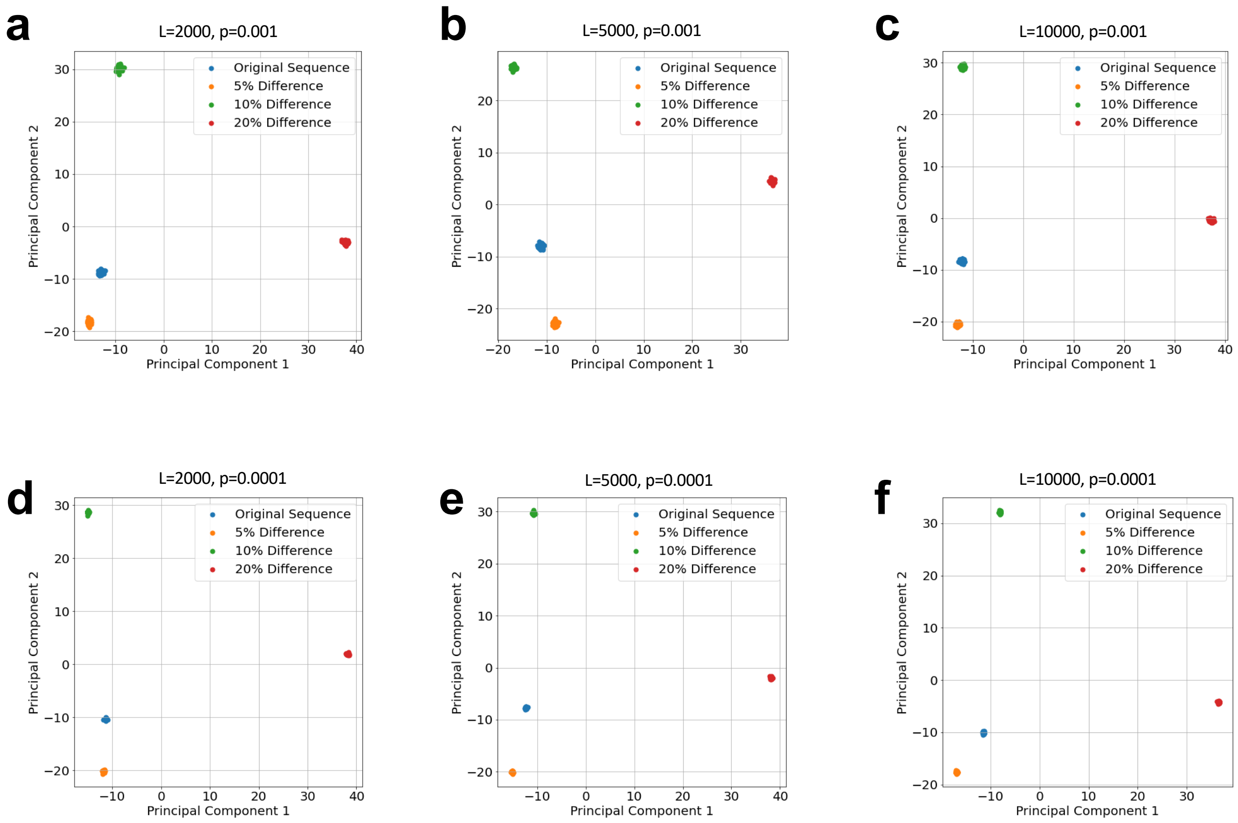

Figure S1. Clustering of Sequences with different starting genomes. In these experiments, we cluster and apply the PCA analysis on sequences generated by different genomes. We first generate a sequence with length L labeled blue (genome U), and then we generate three sequences that are $95\%, 90\%, 80\%$ similar to the blue sequence (i.e., $5\%, 10\%, 20\%$ different from the blue sequence) labeled orange (genome V), green (genome W) and red (genome Z) respectively. Then we add noise on these four sequences $U, V, W,$ and Z with the same probability p and generate 400 sequences (100 each for every genome U, V, W and Z). For noise probability $p = 0.001$, (a)-(c) show the first 2 principal components as a result of applying PCA on the state machine representations of the 400 sequences with $k = 4,\ b = 1, \beta = 0.5$ for different length $L = 2000, 5000, 10000$. For noise probability $p = 0.0001$, (d)-(f) show the first 2 principal components as a result of applying PCA on the state machine representations of the 400 sequences with $k = 4,\ b = 1, \beta = 0.5$ for different length $L = 2000, 5000, 10000$. The implication of this experiment is that if we have a large pool of sequences where sequences have same and low noise or mutation probability but could be generated from multiple genomes (for ex U, V, W and Z), the state machine representation is able to differentiate them using PCA.

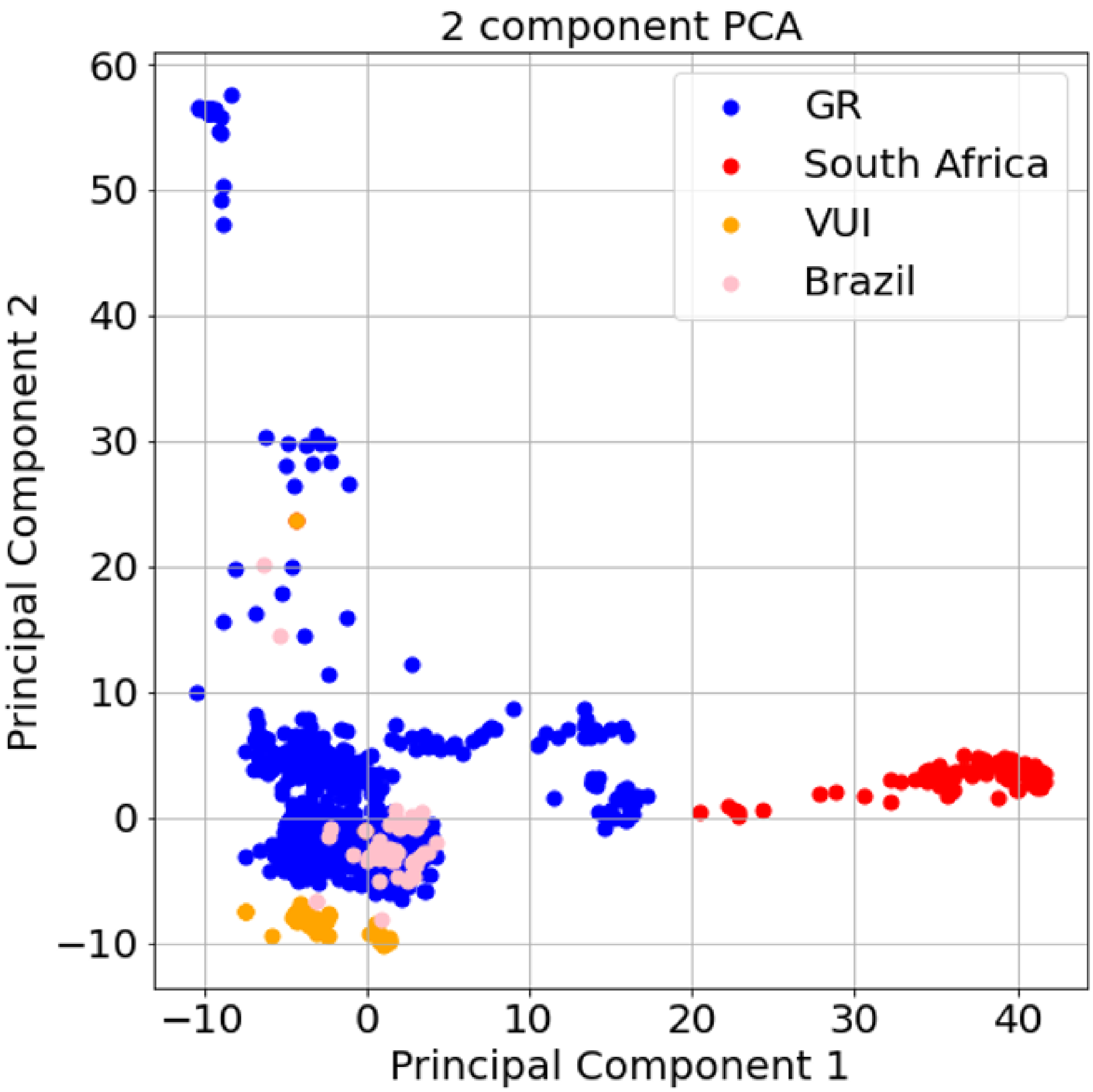

Figure S2. **Clustering of SARS-CoV-2 strains.** PCA Clustering results using our approach for SARS-CoV-2 strains found in UK, South Africa and Brazil alongwith GISAID clade GR.

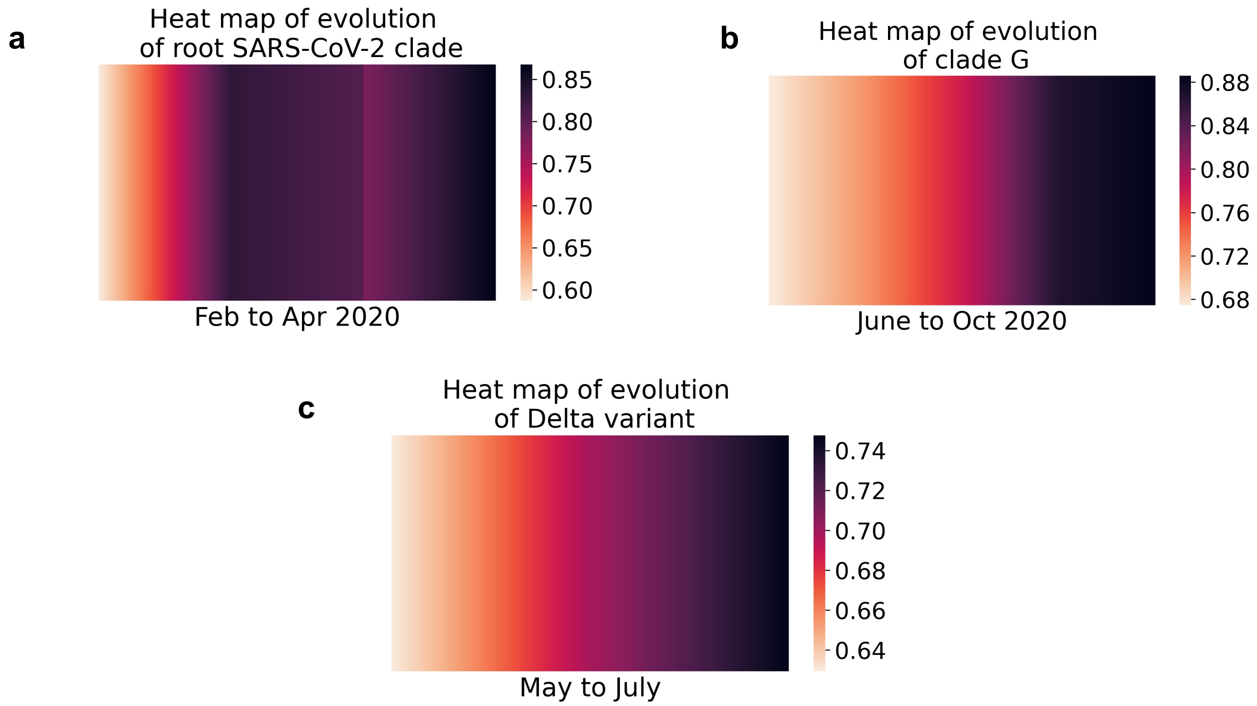

Figure S3. Continuous Evolution heatmaps. (a-c) Heatmaps for continuous evolution of SARS-CoV-2 root clade (Feb to April 2020), clade G (June to Oct 2020) and Delta variant (May to July 2021) respectively.

a

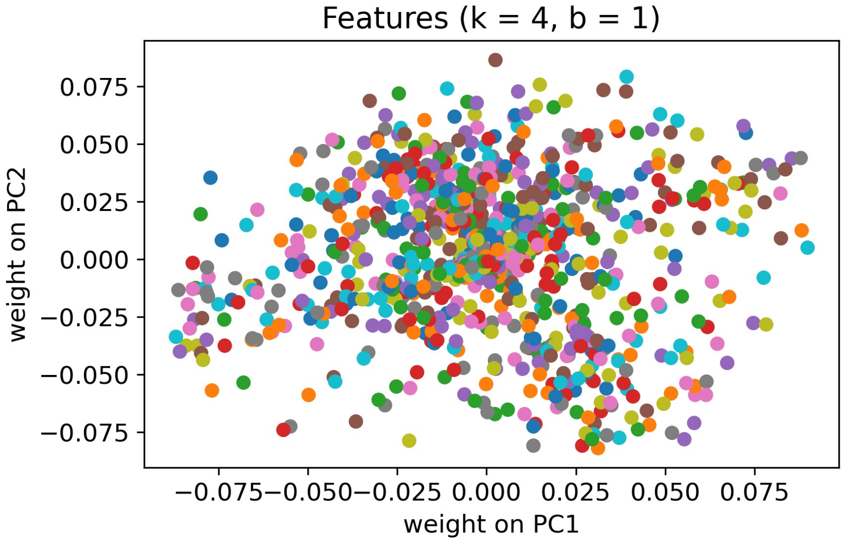

b

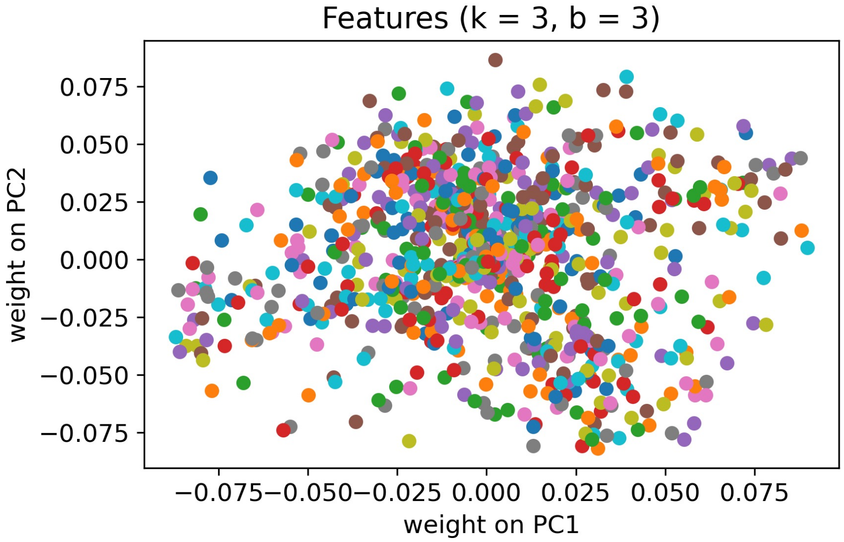

Figure S4. Feature weights on each Principal component. PC1 and PC2 weights for all the features when clades G, L and GR are clustered for **(a)** $k = 4,\ b = 1$. **(b)** $k = 3,\ b = 3$.

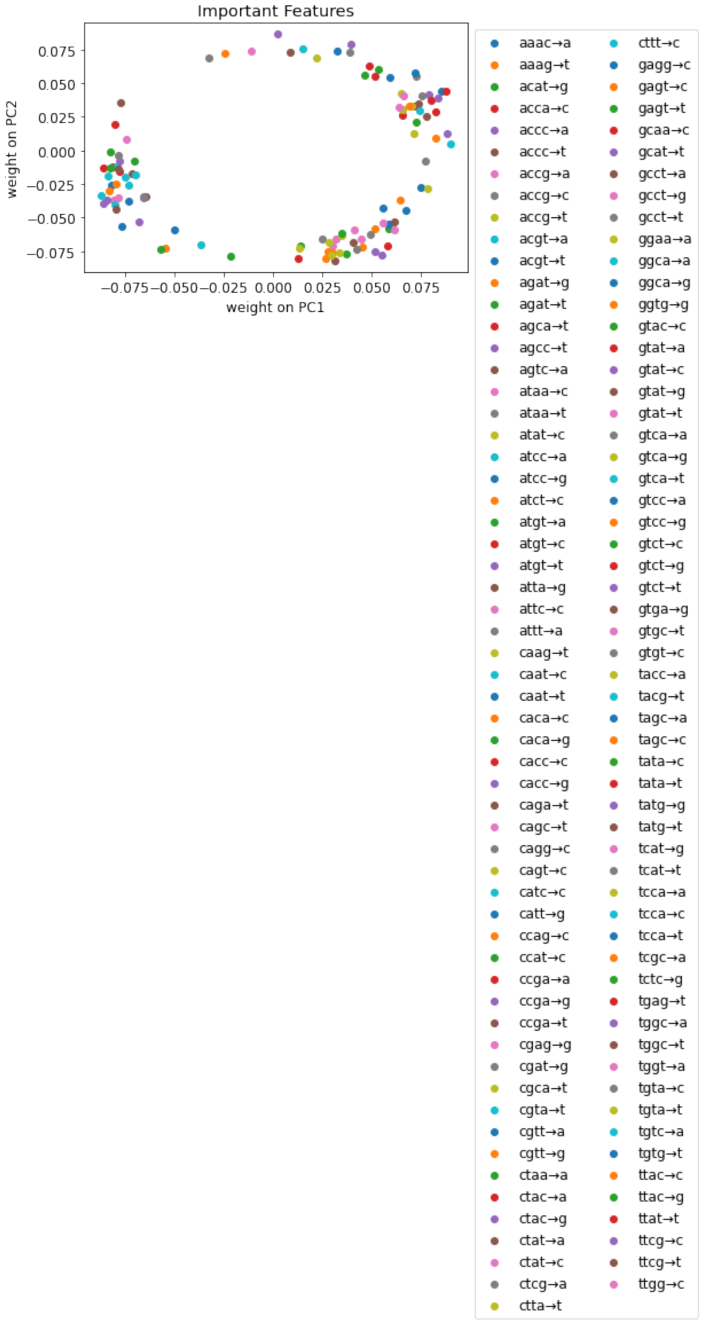

Figure S5. Important Features. Important Features at $k = 4, b = 1$ when clustering clades G, L and GR.

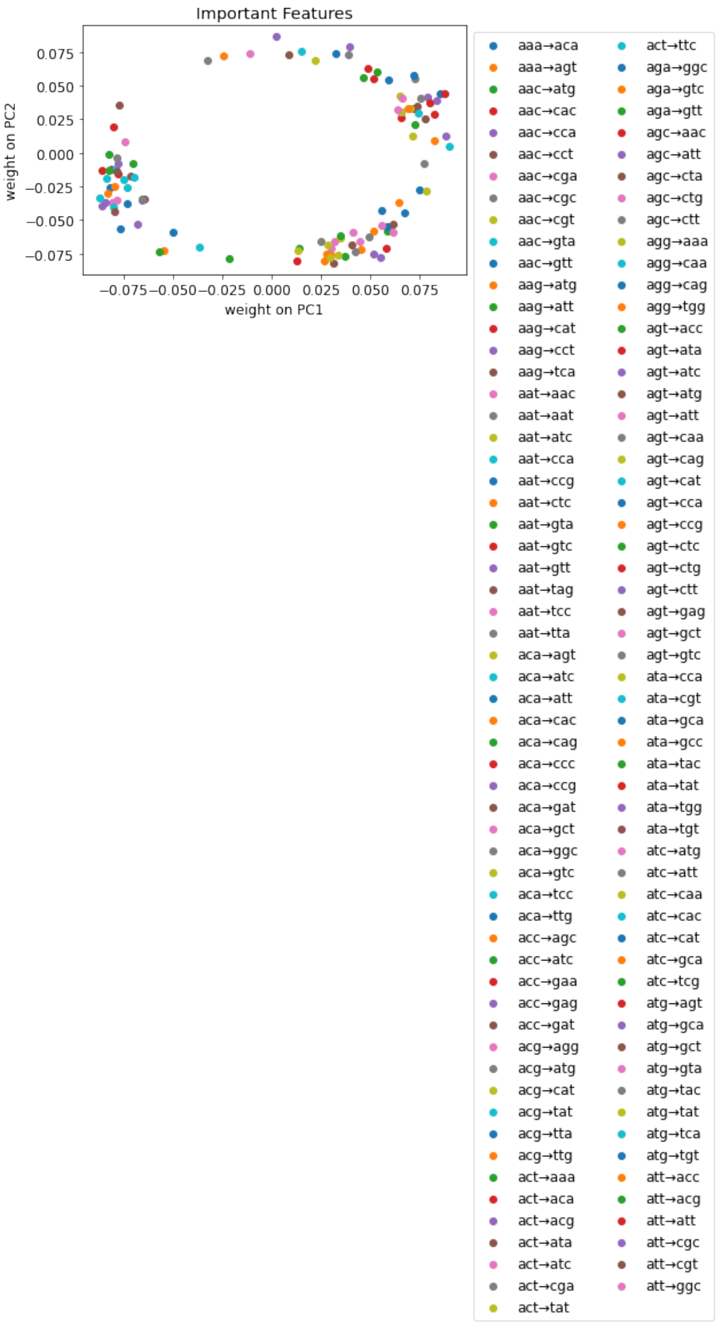

Figure S6. Important Features. Important Features at $k = 3, b = 3$ when clustering clades G, L and GR. The transitions reported in Figure S6 can also be imagined in terms of codons and the corresponding important amino acid transitions are given by $S \to L$, $T \to I$, $T \to S$, $I \to S$, $I \to R$, $N \to V$, $T \to L$, $I \to A$, $N \to P$, $S \to I$, $T \to R$, $T \to T$, $T \to P$, $I \to T$, $K \to L$, $K \to S$, $N \to R$, $N \to L$, $T \to F$, $I \to I$, $T \to Y$, $T \to H$, $M \to S$, $T \to Q$, $I \to P$, $R \to V$, $T \to D$, $I \to H$, $T \to G$, $I \to V$, $N \to I$, $N \to S$, $S \to P$, $K \to I$, $I \to Y$, $T \to E$.
